# Weighted almost convergence and related infinite matrices

**DOI:** 10.1186/s13660-017-1600-z

**Published:** 2018-01-10

**Authors:** Syed Abdul Mohiuddine, Abdullah Alotaibi

**Affiliations:** Operator Theory and Applications Research Group, Department of Mathematics, Faculty of Science, King Abdulaziz University, P.O. Box 80203, Jeddah, 21589 Saudi Arabia

**Keywords:** 46A45, 40G99, 40C05, almost convergence, weighted almost convergence, regular matrix

## Abstract

The purpose of this paper is to introduce the notion of weighted almost convergence of a sequence and prove that this sequence endowed with the sup-norm ${\Vert \cdot \Vert } _{\infty}$ is a BK-space. We also define the notions of weighted almost conservative and regular matrices and obtain necessary and sufficient conditions for these matrix classes. Moreover, we define a weighted almost *A*-summable sequence and prove the related interesting result.

## Introduction and preliminaries

Let *ω* denote the space of all complex sequences $s=(s_{j})_{j=0}^{\infty}$ (or simply write $s=(s_{j})$). Any vector subspace of *ω* is called a sequence space. By $\mathbb {N}$ we denote the set of natural numbers, and by $\mathbb {R}$ the set of real numbers. We use the standard notation $\ell_{\infty}$, *c* and $c_{0}$ to denote the sets of all bounded, convergent and null sequences of real numbers, respectively, where each of the sets is a Banach space with the sup-norm $\Vert . \Vert _{\infty}$ defined by $\Vert s \Vert _{\infty}=\sup_{j\in\mathbb {N}} \vert s_{j} \vert $. We write the space $\ell_{p}$ of all absolutely *p*-summable series by

$$\ell_{p}= \Biggl\{ s\in\omega:\sum_{j=0}^{\infty} \vert s_{j} \vert ^{p}< \infty\ (1\leq p< \infty) \Biggr\} . $$

Clearly, $\ell_{p}$ is a Banach space with the following norm:

$$\Vert s \Vert _{p}= \Biggl(\sum_{j=0}^{\infty} \vert s_{j} \vert ^{p} \Biggr)^{1/p}. $$

For $p=1$, we obtain the set $l_{1}$ of all absolutely summable sequences. For any sequence $s=(s_{j})$, let $s^{[n]}=\sum_{j=0}^{n}s_{j}e_{j}$ be its *n*-section, where $e_{j}$ is the sequence with 1 in place *j* and 0 elsewhere and $e=(1,1,1,\dots)$.

A sequence space *X* is called a BK-space if it is a Banach space with continuous coordinates $p_{j}:X\to\mathbb {C}$, the set of complex fields, and $p_{j}(s)=s_{j}$ for all $s=(s_{j})\in X$ and every $j\in \mathbb {N}$. A BK-space $X\supset\psi$, the set of all finite sequences that terminate in zeros, is said to have AK if every sequence $s=(s_{j})\in X$ has a unique representation $s=\sum_{j=0}^{\infty }s_{j}e_{j}$.

Let *X* and *Y* be two sequence spaces, and let $A=(a_{n,k})$ be an infinite matrix. If, for each $s=(s_{k})$ in *X*, the series

1 $$ A_{n}s=\sum_{k}a_{n,k}s_{k}= \sum_{k=0}^{\infty}a_{n,k}s_{k} $$

converges for each $n\in\mathbb {N}$ and the sequence $As=(A_{n}s)$ belongs to *Y*, then we say that matrix *A* maps *X* into *Y*. By the symbol $(X,Y)$ we denote the set of all such matrices which map *X* into *Y*. The series in (1) is called *A*-*transform* of *s* whenever the series converges for $n=0,1,\ldots $ . We say that $s=(s_{k})$ is *A*-*summable* to the limit *λ* if $A_{n}s$ converges to *λ* ($n\to\infty$).

The sequence $s=(s_{k})$ of $\ell_{\infty}$ is said to be almost convergent, denoted by *f*, if all of its Banach limits [1] are equal. We denote such a class by the symbol *f*, and one writes $f\mbox{-}\lim s =\lambda$ if *λ* is the common value of all Banach limits of the sequence $s=(s_{k})$. For a bounded sequence $s=(s_{k})$, Lorentz [2] proved that $f\mbox{-}\lim s =\lambda $ if and only if

$$\lim_{k\to\infty}\frac{s_{m}+s_{m+1}+\cdots+s_{m+k}}{k+1}=\lambda $$

uniformly in *m*. This notion was later used to (i) define and study conservative and regular matrices [3]; (ii) introduce related sequence spaces derived by the domain of matrices [4–6]; (iii) study some related matrix transformations [7–9]; (iv) define related sequence spaces derived as the domain of the generalized weighted mean and determine duals of these spaces [10, 11]. As an extension of the notion of almost convergence, Kayaduman and Şengönül [12, 13] defined Cesàro and Riesz almost convergence and established related core theorems. The almost strongly regular matrices for single sequences were introduced and characterized [14], and for double sequences they were studied by Mursaleen [15] (also refer to [16–19]). As an application of almost convergence, Mohiuddine [20] proved a Korovkin-type approximation theorem for a sequence of linear positive operators and also obtained some of its generalizations. Başar and Kirişçi [21] determined the duals of the sequence space *f* and other related spaces/series and investigated some useful characterizations.

We now recall the following result.

### Lemma 1.1

([22])

*Let* *X* *and* *Y* *be BK*-*spaces*. (i) *Then* $(X,Y)\subset B(X,Y)$, *that is*, *every* $A\in(X,Y)$ *defines an operator* ${\mathcal{L}}_{A}\in B(X,Y)$ *by* ${\mathcal{L}}_{A}(x)=Ax$ *for all* $x\in X$, *where* $B(X,Y)$ *denotes the set of all bounded linear operators from* *X* *into* *Y*. (ii) *Then* $A\in(X,\ell_{\infty})$ *if and only if* $\Vert A \Vert _{(X,\ell_{\infty})}=\sup_{n} \Vert A_{n} \Vert _{X}<\infty$. *Moreover*, *if* $A\in(X,\ell _{\infty})$, *then* $\Vert {\mathcal{L}}_{A} \Vert = \Vert A \Vert _{(X,\ell_{\infty})}$.

## Weighted almost convergence

### Definition 2.1

Let $t=(t_{k})$ be a given sequence of nonnegative numbers such that $\liminf_{k} t_{k}>0$ and $T_{m}=\sum_{k=0}^{m-1}t_{k}\neq0$ for all $m\geq1$. Then the bounded sequence $s=(s_{k})$ of real or complex numbers is said to be *weighted almost convergent*, shortly $f(\bar{N})$-convergent, to *λ* if and only if

$$\lim_{m\to\infty}\frac{1}{T_{m}}\sum_{k=r}^{r+m-1}t_{k}s_{k}= \lambda\quad\mbox{uniformly in }r. $$

We shall use the notation $f(\bar{N})$ for the space of all sequences which are $f(\bar{N})$-convergent, that is,

2 $$ f(\bar{N})= \Biggl\{ s\in l_{\infty}:\exists\lambda\in\mathbb {C} \ni \lim _{m\to\infty}\frac{1}{T_{m}}\sum_{k=r}^{r+m-1}t_{k}s_{k}= \lambda\mbox{ uniformly in }r; \lambda=f(\bar{N})\mbox{-}\lim s \Biggr\} . $$

We remark that if we take $t_{k}=1$ for all *k*, then (2) is reduced to the notion of almost convergence introduced by Lorentz [2]. Clearly, a convergent sequence is $f(\bar{N})$-convergent to the same limit, but its converse is not always true.

### Example 2.2

Consider a sequence $s=(s_{k})$ defined by $s_{k}=1$ if *k* is odd and 0 for even *k*. Also, let $t_{k}=1$ for all *k*. Then we see that $s=(s_{k})$ is $f(\bar{N})$-convergent to $1/2$ but not convergent.

### Definition 2.3

The matrix *A* (or a matrix map *A*) is said to be *weighted almost conservative* if $As\in f({\bar {N}})$ for all $s=(s_{k})\in c$. One denotes this by $A\in(c,f({\bar {N}}))$. If $A\in(c,f({\bar{N}}))$ with $f({\bar{N}})\mbox{-}\lim As=\lim s$, then we say that *A* is *weighted almost regular matrix*; one denotes such matrices by $A\in(c,f({\bar{N}}))_{R}$.

### Theorem 2.4

*The space* $f(\bar{N})$ *of weighted almost convergence endowed with the norm* ${\Vert \cdot \Vert } _{\infty}$ *is a BK*-*space*.

### Proof

To prove our results, first we have to prove that $f(\bar{N})$ is a Banach space normed by

3 $$ \Vert s \Vert _{f(\bar{N})}=\sup_{m,r} \bigl\vert \Psi_{m,r}(s) \bigr\vert , $$

where

$$\Psi_{m,r}(s)=\frac{1}{T_{m}}\sum_{k=r}^{r+m-1}t_{k}s_{k}. $$

It is easy to verify that (3) defines a norm on $f(\bar{N})$. We have to show that $f(\bar{N})$ is complete. For this, we need to show that every Cauchy sequence in $f(\bar{N})$ converges to some number in $f(\bar{N})$. Let $(s^{k})$ be a Cauchy sequence in $f(\bar{N})$. Then $(s_{j}^{k})$ is a Cauchy sequence in $\mathbb {R}$ (for each $j=1,2,\dots$). By using the notion of the norm of $f(\bar{N})$, it is easy to see that $(s^{k})\to s$. We have only to show that $s\in f(\bar{N})$.

Let $\epsilon>0$ be given. Since $(s^{k})$ is a Cauchy sequence in $f(\bar{N})$, there exists $M\in\mathbb {N}$ (depending on *ϵ*) such that

$$\bigl\Vert s^{k}-s^{i} \bigr\Vert < \epsilon/3\quad \mbox{for all }k,i>M, $$

which yields

$$\sup_{m,r} \bigl\vert \Psi\bigl(s^{k}-s^{i} \bigr) \bigr\vert < \epsilon/3. $$

Therefore we have $\vert \Psi(s^{k}-s^{i}) \vert <\epsilon/3$. Taking the limit as $m\to\infty$ gives that $\vert \lambda ^{k}-\lambda^{i} \vert <\epsilon/3$ for each *m*, *r* and $k,i>M$, where $\lambda^{k}=f(\bar{N})\mbox{-}\lim_{m} s^{k}$ and $\lambda ^{i}=f(\bar{N})\mbox{-}\lim_{m} s^{i}$. Let $\lambda=\lim_{r\to \infty}\lambda^{i}$. Letting $i\to\infty$, one obtains

4 $$ \bigl\vert \Psi_{mr} \bigl(s^{k}-s^{i} \bigr) \bigr\vert < \epsilon/3\quad\mbox{and}\quad \vert\lambda^{k}- \lambda \vert< \epsilon/3 $$

for each *m*, *r* and $k>M$. Now, for fixed *k*, the above inequality holds. Since $s^{k}\in f(\bar{N})$, for fixed *k*, we get

$$\lim_{m\to\infty}\Psi_{mr}\bigl(s^{k}\bigr)= \lambda^{k} \quad\mbox{uniformly in }r. $$

For given $\epsilon>0$, there exists positive integers $M_{0}$ (independent of *r*, but dependent upon *ϵ*) such that

5 $$ \bigl\vert \Psi_{mr} \bigl(s^{k} \bigr)- \lambda^{k} \bigr\vert < \epsilon/3 $$

for $m>M_{0}$ and for all *r*. It follows from (4) and (5) that

$$\begin{aligned} \bigl\vert \Psi_{mr}(s)-\lambda \bigr\vert =& \bigl\vert \Psi_{mr}(s)- \Psi_{mr} \bigl(s^{k} \bigr)+ \Psi_{mr} \bigl(s^{k} \bigr)-\lambda^{k}+ \lambda^{k}-L \bigr\vert \\ \leq& \bigl\vert \Psi_{mr}(s)-\Psi_{mr} \bigl(s^{k} \bigr) \bigr\vert + \bigl\vert \Psi_{mr} \bigl(s^{k} \bigr)- \lambda^{k} \bigr\vert + \bigl\vert \lambda^{k}-L \bigr\vert < \epsilon. \end{aligned}$$

This proves that $f(\bar{N})$ is a Banach space normed by (3).

Since $c\subset f(\bar{N})\subset l_{\infty}$, there exist positive real numbers *α* and *β* with $\alpha<\beta$ such that $\alpha \Vert s \Vert _{\infty}\leq \Vert s \Vert _{f(\bar{N})}\leq\beta \Vert s \Vert _{\infty}$. That is to say, two norms ${\Vert \cdot \Vert } _{\infty}$ and ${\Vert \cdot \Vert } _{f(\bar{N})}$ are equivalent. It is well known that the spaces *c* and $l_{\infty}$ endowed with the norm ${\Vert \cdot \Vert } _{\infty}$ are BK-spaces, and hence the space $f(\bar{N})$ endowed with the norm ${\Vert \cdot \Vert } _{\infty}$ is also a BK-space. □

We prove the following characterization of weighted almost conservative matrices.

### Theorem 2.5

*The matrix* $A=(a_{n,k})$ *is weighted almost conservative*, *that is*, $A\in(c,f({\bar{N}}))$ *if and only if*

6 $$\begin{aligned}& \sup \Biggl\{ \sum_{k=0}^{\infty} \frac{1}{T_{m}} \Biggl\vert \sum_{n=r}^{r+m-1}t_{n}a_{n,k} \Biggr\vert :m\in\mathbb {Z}^{+} \Biggr\} < \infty; \end{aligned}$$

7 $$\begin{aligned}& \lim_{m\to\infty}\frac{1}{T_{m}}\sum_{n=r}^{r+m-1}t_{n}a_{n,k}= \lambda_{k}\quad\textit{exists }(k=0,1,2,\dots) \textit{ uniformly in }r; \end{aligned}$$

8 $$\begin{aligned}& \lim_{m\to\infty}\frac{1}{T_{m}}\sum_{n=r}^{r+m-1} \sum_{k=0}^{\infty}t_{n}a_{n,k}= \lambda\quad\textit{exists uniformly in }r. \end{aligned}$$

### Proof

Necessity. Let $A\in(c,f({\bar{N}}))$. Since the sequences *e* and $e_{k}$ both are convergent, so *A*-transforms of the sequences $e_{k}$ and *e* belong to $f({\bar{N}})$ and exist uniformly in *r*. It follows that (7) and (8) are valid. Let *r* be any nonnegative integer. One writes

$$\Phi_{mr}(s)=\frac{1}{T_{m}}\sum_{n=r}^{r+m-1}t_{n} \alpha_{n}(s), $$

where

$$\alpha_{n}(s)=\sum_{k=0}^{\infty}a_{n,k}s_{k}. $$

It follows that $\alpha_{n}\in c'$ for all $n\in\mathbb {N}_{0}:=\mathbb {N}\cup\{0\}$ and this yields $\Phi_{mr}\in c'$ ($m\geq 1$). Since $A\in(c,f({\bar{N}}))$,

$$\lim_{m\to\infty}\Phi_{mr}(s)=\Phi(s)\quad \mbox{exists uniformly in }r. $$

It is clear that $(\Phi_{mr}(s))$ is bounded for $s=(s_{k})\in c$ and fixed *r*. Hence, by the uniform boundedness principle, $( \Vert \Phi_{mr} \Vert )$ is bounded. For each $p\in\mathbb {Z}^{+}$ (the positive integers), the sequence $x=(x_{k})$ is defined by

$$ x_{k}= \textstyle\begin{cases} \operatorname{sgn}\sum_{n=r}^{r+m-1}t_{n}a_{n,k}&\mbox{if }0\leq k\leq p,\\ 0&\mbox{if }k>p. \end{cases} $$

Then a sequence $x\in c$, $\Vert x \Vert =1$ and

9 $$ \bigl\vert \Phi_{mr}(x) \bigr\vert =\frac{1}{T_{m}}\sum _{k=0}^{p} \Biggl\vert \sum_{n=r}^{r+m-1}t_{n}a_{n,k} \Biggr\vert . $$

Therefore, we obtain

10 $$ \bigl\vert \Phi_{mr}(x) \bigr\vert \leq \Vert \Phi_{mr} \Vert \Vert x \Vert = \Vert \Phi_{mr} \Vert . $$

Equations (9) and (10) give that

$$ \frac{1}{T_{m}}\sum_{k=0}^{p} \Biggl\vert \sum_{n=r}^{r+m-1}t_{n}a_{n,k} \Biggr\vert \leq \Vert \Phi_{mr} \Vert < \infty, $$

it follows that (6) is valid.

Sufficiency. Let conditions (6)-(8) hold. Let *r* be any nonnegative integer, and let $s_{k}\in c$. Then

$$\begin{aligned} \Phi_{mr}(s) =&\frac{1}{T_{m}}\sum_{n=r}^{r+m-1} \sum_{k=0}^{\infty}t_{n}a_{n,k}s_{k} \\ =&\frac{1}{T_{m}}\sum_{k=0}^{\infty}\sum _{n=r}^{r+m-1}t_{n}a_{n,k}s_{k}, \end{aligned}$$

which gives

$$\bigl\vert \Phi_{mr}(s) \bigr\vert \leq\frac{1}{T_{m}}\sum _{k=0}^{\infty} \Biggl\vert \sum_{n=r}^{r+m-1}t_{n}a_{n,k} \Biggr\vert \Vert s \Vert . $$

It follows from hypothesis (6) that $\vert \Phi_{mr}(s) \vert \leq B_{r} \Vert s \Vert $, where $B_{r}$ is a constant independent of *r*. Thus we have $\Phi_{mr}\in c^{\prime}$ for each $m\geq1$, which gives that a sequence $( \Vert \Phi_{mr} \Vert )$ is bounded for each nonnegative integer *r*. Hypotheses (7) and (8) imply that the limit of $\Phi_{mr}(e_{k})$ and $\Phi_{mr}(e)$ must exist for all nonnegative integers *k* and *r*. Since $\{e,e_{0},e_{1},\dots\}$ is a fundamental set in *c*, it follows from [23, p. 252] that $\lim_{m}\Phi_{mr}(s)=\Phi _{r}(s)$ exists and $\Phi_{r}\in c^{\prime}$. Therefore $\Phi_{r}$ has the following form (see [23, p. 205]):

$$\Phi_{r}(s)=\xi\Biggl(\Phi_{r}(e)-\sum _{k=0}^{\infty}\Phi _{r}(e_{k}) \Biggr)+\sum_{k=0}^{\infty}s_{k} \Phi_{r}(e_{k}), $$

where $\xi=\lim s_{k}$. From (7) and (8), we see that $\Phi_{r}(e_{k})=\lambda_{k}$ for a nonnegative integer *k* and $\Phi_{r}(e)=\lambda$. Therefore, for each $s\in c$ and a nonnegative integer *r*, we have

$$\lim_{m\to\infty}\Phi_{mr}(s)=\Phi(s) $$

with the following expression:

11 $$ \Phi(s)=\xi \Biggl(\lambda-\sum_{k=0}^{\infty} \lambda_{k} \Biggr)+\sum_{k=0}^{\infty}s_{k} \lambda_{k}. $$

Since $\Phi_{mr}\in c^{\prime}$, so it has the representation

12 $$ \Phi_{mr}(s)=\xi \Biggl(\Phi_{mr}(e)-\sum _{k=0}^{\infty}\Phi_{mr}(e_{k}) \Biggr)+ \sum_{k=0}^{\infty}s_{k} \Phi_{mr}(e_{k}). $$

We observe from (11) and (12) that the convergence of $\Phi_{mr}(s)$ to $\Phi(s)$ is uniform since $\lim_{m\to\infty }\Phi_{mr}(e_{k})=\lambda_{k}$ and $\lim_{m\to\infty}\Phi _{mr}(e)=\lambda$ uniformly in *r*. Hence, *A* is a weighted almost conservative matrix. □

In the following theorem, we obtain the characterization of weighted almost regular matrices.

### Theorem 2.6

*The matrix* $A\in(c,f({\bar{N}}))_{R}$ *if and only if*

13 $$\begin{aligned}& \sup \Biggl\{ \sum_{k=0}^{\infty} \frac{1}{T_{m}} \Biggl\vert \sum_{n=r}^{r+m-1}t_{n}a_{n,k} \Biggr\vert :m\in\mathbb {Z}^{+} \Biggr\} < \infty; \end{aligned}$$

14 $$\begin{aligned}& \lim_{m\to\infty}\frac{1}{T_{m}}\sum_{n=r}^{r+m-1}t_{n}a_{n,k}=0 \quad\textit{uniformly in }r~(k\in\mathbb {N}_{0}); \end{aligned}$$

15 $$\begin{aligned}& \lim_{m\to\infty}\frac{1}{T_{m}}\sum_{n=r}^{r+m-1} \sum_{k=0}^{\infty}t_{n}a_{n,k}=1 \quad\textit{uniformly in }r. \end{aligned}$$

### Proof

Necessity. Let $A\in(c,f({\bar{N}}))_{R}$. We see that condition (13) holds by using the fact that *A* is also weighted almost conservative. Take $e_{k},e\in c$. Then *A*-transforms of the sequences $e_{k}$ and *e* are weighted almost convergent to 0 and 1, respectively, since $e_{k}\to0$ and $e\to1$. Hence $e_{k}\in c$ gives condition (14) and $e\in c$ proves the validity of (15).

Sufficiency. Let conditions (13)-(15) hold. It is easy to see that *A* is weighted almost conservative. So, for each $(s_{k})\in c$, $\lim_{m\to\infty}\Phi_{mr}(s)=\Phi(s)$ uniformly in *r*. Thus we obtain from (11) and our hypotheses (13)-(15) that $\Phi(s)=\xi=\lim s_{k}$. This yields *A* is weighted almost regular. □

We now obtain necessary and sufficient conditions for the matrix *A* which transform the absolutely convergent series into the space of weighted almost convergence.

### Theorem 2.7

*The matrix* $A\in(l_{1},f({\bar{N}}))$ *if and only if*

16 $$\begin{aligned}& \sup_{k,m,r} \Biggl\vert \frac{1}{T_{m}}\sum _{n=r}^{r+m-1}t_{n}a_{n,k} \Biggr\vert < \infty, \end{aligned}$$

17 $$\begin{aligned}& \lim_{m\to\infty}\frac{1}{T_{m}}\sum_{n=r}^{r+m-1}t_{n}a_{n,k}= \lambda_{k}\quad\textit{exists for each }k\in\mathbb {N}_{0} \textit{ uniformly in }r. \end{aligned}$$

### Proof

Necessity. Let $A\in(l_{1},f({\bar{N}}))$. Condition (17) follows since $e_{k}\in l_{1}$. Let $\Phi_{mr}$ be a continuous linear functional on $l_{1}$ defined by

$$ \Phi_{mr}(s)=\frac{1}{T_{m}}\sum _{k=0}^{\infty}\sum_{n=r}^{r+m-1}t_{n}a_{n,k}s_{k}. $$

Then we have

$$ \bigl\vert \Phi_{mr}(s) \bigr\vert \leq\sup_{k} \Biggl\vert \frac{1}{T_{m}}\sum_{n=r}^{r+m-1}t_{n}a_{n,k} \Biggr\vert \Vert s \Vert _{1}, $$

which yields

18 $$ \Vert \Phi_{mr} \Vert \leq\sup_{k} \Biggl\vert \frac{1}{T_{m}}\sum_{n=r}^{r+m-1}t_{n}a_{n,k} \Biggr\vert . $$

For any fixed $k\in\mathbb {N}_{0}$, we define a sequence $s=(s_{j})$ by

$$ s_{j}= \textstyle\begin{cases} \operatorname{sgn}\sum_{n=r}^{r+m-1}t_{n}a_{n,k}&\mbox{if }j=k,\\ 0&\mbox{if }j\neq k. \end{cases} $$

Then we have $\Vert s \Vert _{1}=1$ and

$$\bigl\vert \Phi_{mr}(s) \bigr\vert = \Biggl\vert \frac{1}{T_{m}}\sum _{n=r}^{r+m-1}t_{n}a_{n,k}s_{k} \Biggr\vert = \Biggl\vert \frac{1}{T_{m}}\sum_{n=r}^{r+m-1}t_{n}a_{n,k} \Biggr\vert \Vert s \Vert _{1}, $$

so

19 $$ \Vert \Phi_{mr} \Vert \geq\sup_{k} \Biggl\vert \frac{1}{T_{m}}\sum_{n=r}^{r+m-1}t_{n}a_{n,k} \Biggr\vert . $$

We obtain from (18) and (19) that

$$ \Vert \Phi_{mr} \Vert =\sup_{k} \Biggl\vert \frac{1}{T_{m}}\sum_{n=r}^{r+m-1}t_{n}a_{n,k} \Biggr\vert . $$

Since $A\in(l_{1},f({\bar{N}}))$, for any $s\in l_{1}$, we have

20 $$ \sup_{m,r} \bigl\vert \Phi_{mr}(s) \bigr\vert = \sup _{m,r} \Biggl\vert \frac{1}{T_{m}}\sum_{k=0}^{\infty} \sum_{n=r}^{r+m-1}t_{n}a_{n,k}s_{k} \Biggr\vert < \infty. $$

By using the uniform boundedness theorem, Equation (20) becomes

$$ \sup_{m,r} \Vert \Phi_{mr} \Vert =\sup _{k,m,r} \Biggl\vert \frac{1}{T_{m}}\sum_{n=r}^{r+m-1}t_{n}a_{n,k} \Biggr\vert < \infty. $$

This proves the validity of (16).

Sufficiency. Let conditions (16) and (17) hold, and let $s=(s_{k})\in l_{1}$. In virtue of these conditions, we see that

21 $$ \lim_{m\to\infty}\frac{1}{T_{m}}\sum_{k=0}^{\infty} \sum_{n=r}^{r+m-1}t_{n}a_{n,k}s_{k}= \sum_{k=0}^{\infty}\lambda_{k}s_{k} \quad\mbox{uniformly in }r, $$

it also converges absolutely. Furthermore, $\frac{1}{T_{m}}\sum_{k=0}^{\infty}\sum_{n=r}^{r+m-1}t_{n}a_{n,k}s_{k}$ converges absolutely for each *m* and *r*.

Let $\epsilon>0$ be given. Then there exists $k_{0}\in\mathbb {N}$ such that

22 $$ \sum_{k>k_{0}} \vert s_{k} \vert< \epsilon. $$

By condition (17), we can find some $m_{0}\in\mathbb {N}$ such that

23 $$ \Biggl\vert \sum_{k\leq k_{0}} \Biggl[\frac{1}{T_{m}}\sum _{n=r}^{r+m-1}t_{n}a_{n,k}- \lambda_{k} \Biggr]s_{k} \Biggr\vert < \epsilon $$

for all $m>m_{0}$ uniformly in *r*. Now

24 $$\begin{aligned} \Biggl\vert \sum_{k=0}^{\infty} \Biggl[ \frac{1}{T_{m}}\sum_{n=r}^{r+m-1}t_{n}a_{n,k}- \lambda_{k} \Biggr]s_{k} \Biggr\vert \leq& \Biggl\vert \sum _{k\leq k_{0}} \Biggl[\frac{1}{T_{m}}\sum _{n=r}^{r+m-1}t_{n}a_{n,k}- \lambda_{k} \Biggr]s_{k} \Biggr\vert \\ &{}+\sum_{k>k_{0}} \Biggl\vert \frac{1}{T_{m}}\sum _{n=r}^{r+m-1}t_{n}a_{n,k}- \lambda_{k} \Biggr\vert \vert s_{k} \vert \end{aligned}$$

for all $m>m_{0}$ uniformly in *r*. By using Equations (22) and (23) and our hypotheses in the above inequality, we see that (21) holds, and hence the sufficiency part. □

### Theorem 2.8

*If the matrix* *A* *in* $(l_{1},f({\bar {N}}))$, *then* $\Vert {\mathcal{L}}_{A} \Vert = \Vert A \Vert $.

### Proof

Let $A\in(l_{1},f({\bar{N}}))$. Then we have

$$\bigl\Vert {\mathcal{L}}_{A}(s) \bigr\Vert =\sup _{m,r} \Biggl\vert \frac {1}{T_{m}}\sum_{k=0}^{\infty} \sum_{n=r}^{r+m-1}t_{n}a_{n,k}s_{k} \Biggr\vert \leq\sup_{m,r}\sum_{k=0}^{\infty} \Biggl\vert \frac {1}{T_{m}}\sum_{n=r}^{r+m-1}t_{n}a_{n,k} \Biggr\vert \vert s_{k} \vert , $$

which gives $\Vert {\mathcal{L}}_{A}(s) \Vert \leq \Vert A \Vert \Vert s \Vert _{1}$. This implies that $\Vert {\mathcal{L}}_{A} \Vert \leq \Vert A \Vert $. Also, ${\mathcal{L}}_{A}\in B(l_{1},f({\bar{N}}))$ gives

$$\bigl\Vert {\mathcal{L}}_{A}(s) \bigr\Vert = \Vert As \Vert \leq \Vert {\mathcal{L}}_{A} \Vert \Vert s \Vert _{1}. $$

Taking $s=(e_{k})$ and using the fact that $\Vert e_{k} \Vert _{1}=1$ ∀*k*, one obtains $\Vert A \Vert \leq \Vert {\mathcal{L}}_{A} \Vert $. Hence we conclude that $\Vert {\mathcal{L}}_{A} \Vert = \Vert A \Vert $. □

### Definition 2.9

Let $t=(t_{k})_{k\in\mathbb {N}}$ be a given sequence of nonnegative numbers such that $\liminf_{k} t_{k}>0$ and $T_{m}=\sum_{k=0}^{m-1}t_{k}\neq0$ for all $m\geq1$. A sequence $s=(s_{k})$ is said to be *weighted almost* *A-summable* to $\lambda\in\mathbb {C}$ if the *A*-transform of sequence $s=(s_{k})$ is weighted almost convergent to *λ*; equivalently, we can write

$$\lim_{m}\sigma_{mr}(s)=\lambda\quad\mbox{uniformly in }r, $$

where

$$\sigma_{mr}(s)=\frac{1}{T_{m}}\sum_{n=r}^{r+m-1} \sum_{k=0}^{\infty }t_{n}a_{n,k}s_{k}. $$

In the applications of summability theory to function theory, it is important to know the region in which $S=(S_{k}(z))$, the sequence of partial sums of the geometric series is *A*-summable to $\frac {1}{1-z}$ for a given matrix *A*. In the following theorem, we find the region in which *S* is weighted almost *A*-summable to $\frac{1}{1-z}$.

### Theorem 2.10

*Let* $A=(a_{n,k})$ *be a matrix such that* (15) *holds*. *The sequence* $(S_{k}(z))$ *is weighted almost* *A*-*summable to* $\frac{1}{1-z}$ *if and only if* $z\in R$, *where*

$$ R= \Bigl\{ z= \bigl(z^{k} \bigr):\lim_{m} \sigma_{mr}(z)=0 \textit{ uniformly in }r \Bigr\} . $$

### Proof

One writes

$$\begin{aligned} \sigma_{mr} =&\frac{1}{T_{m}}\sum_{n=r}^{r+m-1} \sum_{k=0}^{\infty}t_{n}a_{n,k}S_{k}(z) \\ =&\frac{1}{T_{m}}\sum_{n=r}^{r+m-1}\sum _{k=0}^{\infty}t_{n}a_{n,k} \frac{1-z^{k+1}}{1-z} \\ =&\frac{1}{(1-z)T_{m}}\sum_{n=r}^{r+m-1}\sum _{k=0}^{\infty}t_{n}a_{n,k}- \frac{z}{(1-z)T_{m}}\sum_{n=r}^{r+m-1}\sum _{k=0}^{\infty}t_{n}a_{n,k}z^{k}. \end{aligned}$$

Taking the limit as $m\to\infty$ in the above equality and using condition (15), one obtains

$$\lim_{m\to\infty}\sigma_{mr}=\frac{1}{1-z} \quad \mbox{uniformly in }r $$

if and only if $z\in R$. This completes the proof. □
